# Systematic omics analysis identifies CCR6 as a therapeutic target to overcome cancer resistance to EGFR inhibitors

**DOI:** 10.1016/j.isci.2024.109448

**Published:** 2024-03-07

**Authors:** Eun-Ji Kwon, Hyuk-Jin Cha, Haeseung Lee

**Affiliations:** 1 College of Pharmacy, Seoul National University, Seoul 08826, Republic of Korea; 2 Department of Pharmacy, College of Pharmacy and Research Institute for Drug Development, Pusan National University, Busan 46241, Republic of Korea

**Keywords:** Integrative aspects of cell biology, Cancer systems biology, Cancer, Omics

## Abstract

Epidermal growth factor receptor inhibitors (EGFRi) have exhibited promising clinical outcomes in the treatment of various cancers. However, their widespread application has been limited by low patient eligibility and the emergence of resistance. Leveraging a multi-omics approach (>1000 cancer cell lines), we explored molecular signatures linked to EGFRi responsiveness and found that expression signatures involved in the estrogen response could recapitulate cancer cell dependency on EGFR, a phenomenon not solely attributable to EGFR-activating mutations. By correlating genome-wide function screening data with EGFRi responses, we identified chemokine receptor 6 (CCR6) as a potential druggable target to mitigate EGFRi resistance. In isogenic cell models, pharmacological inhibition of CCR6 effectively reversed acquired EGFRi resistance, disrupting mitochondrial oxidative phosphorylation, a cellular process commonly associated with therapy resistance. Our data-driven strategy unveils drug-response biomarkers and therapeutic targets for resistance, thus potentially expanding EGFRi applicability and efficacy.

## Highlights

• DepMap data analysis reveals molecular signatures of cell response to EGFR inhibitor
• Estrogen response signatures indicate cancer cell dependency on EGFR inhibitors
• Chemokine receptor 6 is a potential target to combat EGFR inhibitor resistance

## Graphical abstract

**Figure:**
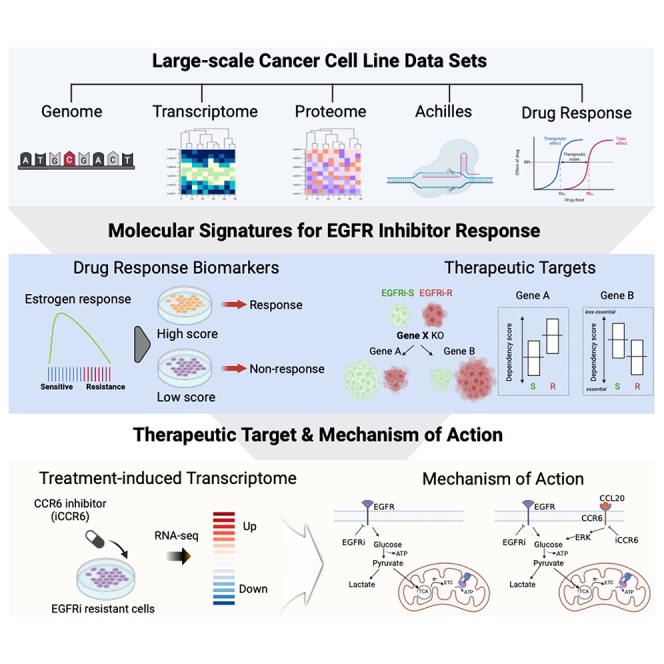

## Introduction

Despite the promising clinical outcomes of targeted therapeutics, the overall eligibility for these treatments remains relatively low, representing only around 10% of cancer patients in the USA.^1^ In particular, a selected group of patients, referred to as ‘a lucky few’,^2^ who have specific mutations in the epithelial growth factor receptor (EGFR) gene,^3^ are candidates for treatment with EGFR inhibitor (EGFRi). These mutations induce continuous activation of the EGFR pathways, making the cancer highly dependent on EGFR activity for its survival, a phenomenon known as ‘oncogene addiction’.^4^ In addition, while initial success has been achieved with EGFRi, most patients eventually experience disease progression due to the emergence of drug resistance over time. Consequently, recent research efforts have focused on resolving not only acquired resistance^5^ but also the low eligibility rate for EGFRi treatment.^6^

Cancer cell lines (CCLs) have been extensively utilized in cancer research investigating therapeutic targets or drug-response biomarkers.^7^^,^^8^ More than 1000 different CCLs established from various cancer types have been accumulated, effectively representing the diversity of human cancers.^9^^,^^10^ Most notably, large-scale initiatives such as the Cancer Cell Line Encyclopedia (CCLE),^7^^,^^8^ the Cancer Therapeutic Response Portal (CTRP),^11^^,^^12^ and the Genomics of Drug Sensitivity in Cancer (GDSC)^13^ have produced vast amounts of publicly accessible data on the genomic, transcriptomic, and proteomic profiles of hundreds of CCLs, along with their responses to chemical perturbation. Advancements in genome-wide loss-of-function platforms based on RNA silencing or CRISPR-Cas9 have also facilitated the construction of large-scale genetic perturbation screening databases for extensive collections of CCLs, including transcriptome profile (LINCS^14^) and vulnerability profiles (the projects DRIVE^15^ and Achilles^16^). These gene-dependency data enable the systematic identification of genes essential for the survival of specific cancer cells.^14^^,^^17^^,^^18^

By leveraging these large-scale CCL datasets, ongoing research efforts have sought to integrate multi-omics data with phenotypic data for cancer cells to identify molecular signatures associated with cancer cell responses to anticancer therapies.^19^ An integrated strategy utilizing chemical and genetic perturbation data, centered around the common transcriptome profile of therapy-resistant cancer cells, has identified specific small molecules capable of inducing targeted cell death through lipid peroxidation (i.e., ferroptosis), as validated experimentally.^20^ This data-driven approach has also led to the identification of gene expression signatures related to drug responsiveness,^21^^,^^22^^,^^23^^,^^24^ and drug repositioning by integrating transcriptome signatures of chemical and genetic perturbation.^25^^,^^26^^,^^27^ These strategies are anticipated to overcome the limitations of current targeted therapeutics, including the low patient eligibility for drugs such as EGFRi, by identifying accompanying drugs with EGFRi based on molecular and phenotypic features observed in CCLs, thus effectively broadening the range of treatment options.^28^^,^^29^

In this study, we exploited these extensive CCL datasets to enhance our understanding of EGFRi responsiveness. Our investigation provides valuable insights into the transcriptomic and proteomic signatures linked to EGFRi sensitivity, offering a viable alternative to the traditional use of the EGFR mutational status as the exclusive response biomarker. Moreover, by correlating genome-wide loss-of-function data with drug response data, we identified chemokine receptor 6 (CCR6) as a potential target for increasing the sensitivity of EGFRi-resistant CCLs. Notably, the pharmacological inhibition of CCR6 effectively reversed acquired resistance to EGFRi in cancer cells and disrupted resistance-associated glucose metabolism.

## Results

### EGFR oncogenic mutations representing EGFRi sensitivity

**Figure 1:**
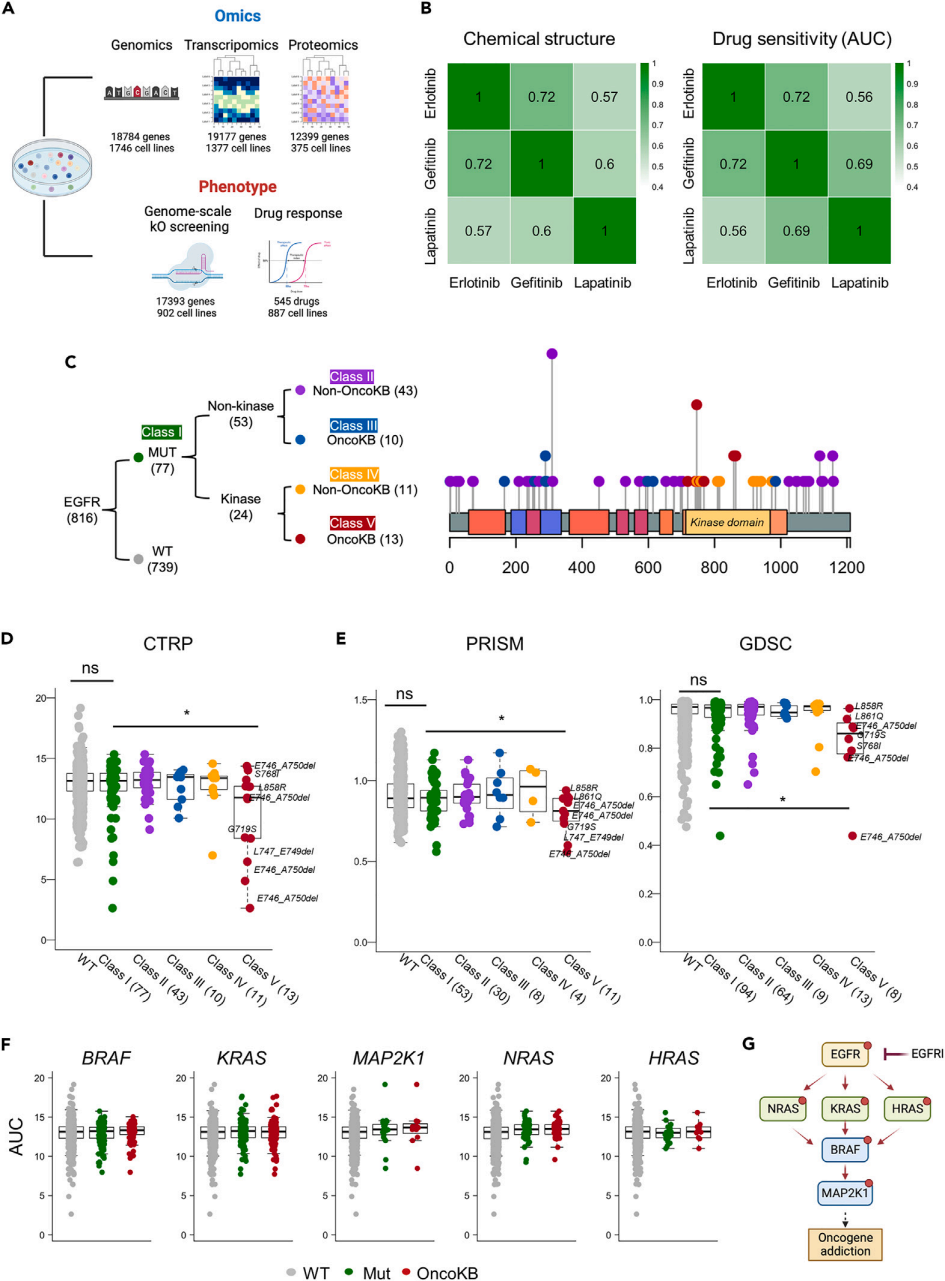
Relationship between EGFR mutation and EGFRi sensitivity (A) Schematic illustration depicting the number of CCLs and genes included in the omics and phenotypic datasets employed for this study. (B) Heatmaps showing correlation coefficients, illustrating similarities in chemical structure (left) and drug responsiveness (right) between EGFRi. (C) EGFR mutation classes categorized by their location and oncogenic effect (left). The oncogenic effects of specific mutations were determined using annotations from the OncoKB database. Lollipop plot depicts the locations and frequencies of observed EGFR mutations in CCLs (right). (D and E) AUC values representing EGFRi drug responsiveness in CCL subgroups, obtained from (D) the CTRP, (E) GDSC, and PRISM. (F) EGFRi drug responsiveness AUC values in CCL categorized by the mutational status of genes involved in mediating EGFR signaling. (G) Schematic diagram showing the genes involved in mediating EGFR signaling. Data are presented as the mean ± standard deviation (SD) of independent experiments. Statistical significance was assessed using p value threshold of 0.05. Asterisks denote significance levels: ∗p < 0.05, ∗∗p < 0.01, ∗∗∗p < 0.001, ∗∗∗∗p < 0.0001; n.s. denotes non-significant.

To gain molecular insights into cancer cell responsiveness to EGFRi, we employed comprehensive CCL datasets containing both omics and phenotypic information (Figure 1A). We specifically utilized three EGFRi drugs—erlotinib, gefitinib, and lapatinib—which are available in CTRP, share similar chemical structures, and elicit comparable cellular responses (Figures S1A and S1B). Because EGFR oncogenic mutations serve as indicators of sensitivity to EGFRi,^30^ we initially examined the association between the mutational status of the *EGFR* gene in the CCLs and its responsiveness to EGFRi. Of the 816 CCLs analyzed, 92 were identified as harboring EGFR mutations leading to protein changes. Interestingly, the overall mutation status showed no significant relationship with cellular sensitivity to EGFRi. This observation was replicated in the other pharmacogenomic databases GDSC and PRISM (Figure S1B).

Given that EGFRi treatment is primarily employed on NSCLC patients with activating mutations within the kinase domain of *EGFR*, specifically in exon 18–21,^6^^,^^31^ we categorized EGFR mutations into five classes based on their specific location and oncogenic effects as annotated by OncoKB^32^ (Figure 1C). These five classes encompassed the entire spectrum of EGFR mutations, spanning from mutations covering the entire EGFR gene (Class I, n = 92), mutations outside the kinase domain without annotated oncogenic effects (Class II, n = 43), mutations outside the kinase domain with annotated oncogenic effects (Class III, n = 10), mutations within the kinase domain without annotated oncogenic effects (Class IV, n = 11), and mutations within the kinase domain with annotated oncogenic effects (Class V, n = 13) (Figure 1C). Remarkably, only mutations falling into Class V showed a significant correlation with cellular sensitivity to EGFRi in CTRP (Figure 1D), as well as in PRISM and GDSC (Figure 1E; Table S1). This indicates that only a few specific subsets of the mutations that drive oncogene addiction^4^ have value as a therapeutic biomarker for EGFRi therapy.

We also explored the mutation status of genes involved in the transmission of EGFR signaling (*BRAF, KRAS*, *MAP2K1, NRAS*, and *HRAS*) and found no significant association with EGFRi sensitivity (Figure 1F) because their oncogenic mutations circumvent the effect of EGFRi (Figure 1G). Consequently, as described previously,^6^ relying solely on the EGFR mutational status as an indicator of EGFRi efficacy would be insufficient. This is why only a few designated EGFR mutations, such as L858R, S768I, L861Q, and G719S, are employed to guide EGFRi therapy decisions in oncology clinics.^6^

### Gene signatures correlated with the EGFRi sensitivity

**Figure 2:**
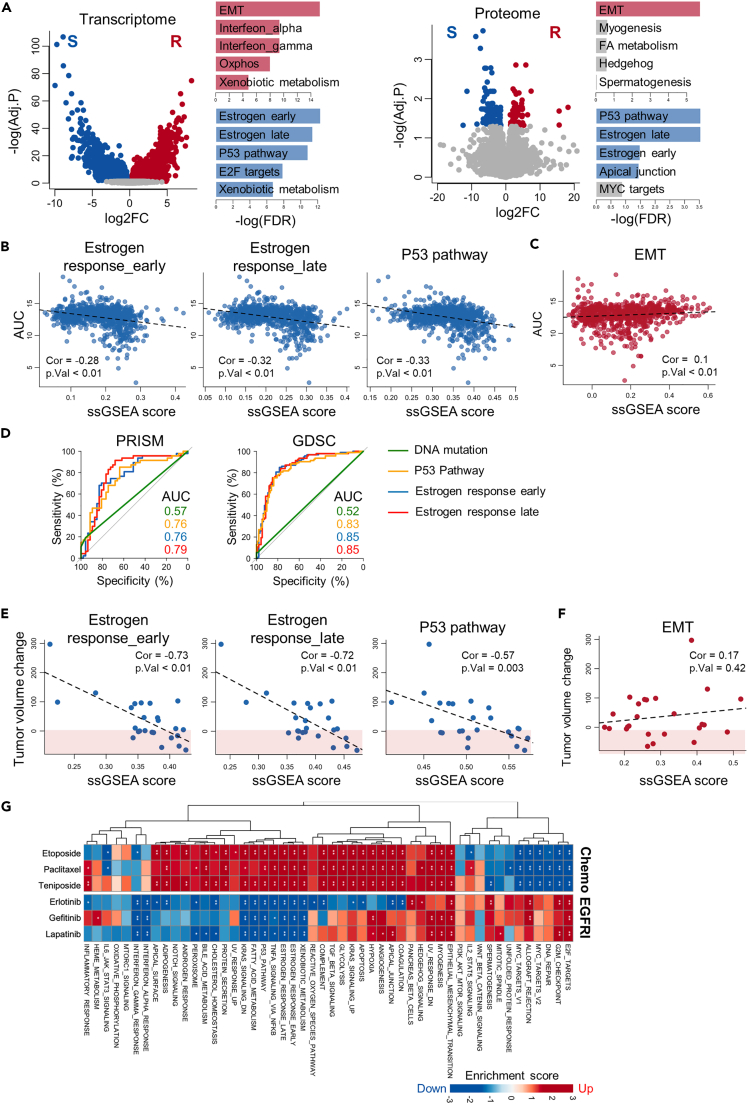
Omics signatures associated with EGFRi responsiveness (A) Volcano plot illustrating DEGs (left) and DEPs (right) in EGFRi-R vs. EGFRi-S cell groups. The top five MSigDB hallmark gene sets enriched in DEGs and DEPs are displayed next to the plot, with upregulated sets shown in red and downregulated sets in blue bars. Gene sets with FDR exceeding 0.05 are shown in gray. (B and C) Association between drug response AUC values and transcriptomic signatures for (B) the estrogen response (early and late) P53 pathway, and (C) EMT signature scores across CCLs. (D) The ROC curve showing the true-positive rates against the false-positive rates for predicting EGFRi-S cells. The ROC curve of random expectations is shown by a gray line. (E and F) Association between drug response AUC values and transcriptomic signatures for (B) the estrogen response (early and late), P53 pathway, and (C) EMT signature scores across PDX models. (G) Heatmap illustrating the enrichment score for MSigDB hallmark gene sets in DEGs of resistant cells compared to sensitive cells to the indicated drugs, quantified through GSEA. Data are presented as the mean ± standard deviation (SD) of independent experiments. Statistical significance was assessed using p value threshold of 0.05. Asterisks denote significance levels: ∗p < 0.05, ∗∗p < 0.01, ∗∗∗p < 0.001, ∗∗∗∗p < 0.0001; n.s. denotes non-significant.

To explore additional molecular factors contributing to the sensitivity observed in CCLs lacking oncogenic mutations, we conducted a comparative analysis utilizing the transcriptomic and proteomic profiles of two distinct subsets of CCLs that differed in their response to EGFRi as determined by the highest and lowest deciles of the AUC: EGFRi-R and EGFRi-S, respectively (Figure S2A). We conducted functional enrichment analysis on differentially expressed genes and proteins between the EGFRi-R and EGFRi-S groups, using MSigDB hallmark gene sets. This analysis showed that genes associated with the epithelial–mesenchymal transition (EMT) were consistently upregulated in the EGFRi-R group both at the gene (Figure 2A, top left) and protein expression levels (Figure 2A, top right; Table S2). This finding aligns with the well-established association between the EMT and resistance against various drugs,^26^^,^^33^ including EGFRi.^34^ In contrast, the EGFRi-S group showed upregulation of genes involved in both the early and late responses to estrogen, as well as genes in the P53 signaling pathway (Figure 2A). In addition to the clear crosstalk between the estrogen receptor (ER) and EGFR,^35^ the estrogen response is also potentiated by ER phosphorylation through EGFR activation.^36^ In addition, genes within the P53 pathway are known to contribute to primary sensitivity to EGFRi.^37^ These observations suggest that the estrogen response and/or P53 pathway are a possible index of EGFR activity.

We next employed single-sample GSEA (ssGSEA) to assign an expression-based activity score for the estrogen response, P53 pathway, and EMT signatures to each CCL and assessed their correlation with EGFRi responsiveness. The enrichment scores for the estrogen response and P53 pathway had a significant negative correlation with AUC values across the remaining 80% of the CCLs, excluding the EGFRi-R and EGFRi-S cell groups (Figures 2B and S2B). This means that the higher expression levels of genes associated with these signatures are linked to higher sensitivity to EGFRi. However, the EMT signature exhibited no significant correlation with EGFRi responsiveness (Figures 2C and S2C), indicating that only highly resistant CCLs in the EGFRi-R group have a pronounced EMT signature. In addition, we compared the predictive performance of these transcriptomic signatures for EGFRi sensitivity to that of EGFR mutational status using independent databases GDSC and PRISM. Remarkably, the estrogen response (AUROC: 0.85) followed by the P53 pathway signatures (AUROC: 0.82) outperformed EGFR mutational status (AUROC: 0.52) (Figure 2D). This highlights the potential of these signatures as robust predictors of EGFRi sensitivity, surpassing the conventional reliance on the EGFR mutational status as the sole response biomarker. To further confirm the associations between these signatures and EGFRi sensitivity, we retrieved large-scale drug efficacy screening data with patient-derived tumor xenografts (PDX).^38^ Consistent with our initial predictions, PDX tumors with elevated transcriptomic signatures of the estrogen response or the P53 pathway experienced a notable reduction in tumor size following EGFRi treatment (Figure 2E). Conversely, tumors with a high EMT signature demonstrated resistance to this treatment (Figure 2F).

To discern whether these observed expression signatures exclusively indicated resistance to EGFRi or rather reflected a general resistance to broad-spectrum anticancer drugs, we explored transcriptomic signatures associated with responsiveness to conventional chemotherapeutic agents such as etoposide, paclitaxel, and teniposide (referred to as Chemo). Remarkably, the pattern for the transcriptomic signatures associated with Chemo responsiveness differed from those associated with EGFRi responsiveness, except for EMT (Figure 2G). Unlike the signatures related to EGFRi sensitivity, gene signatures linked to active cell cycle processes, including the G2/M checkpoint and targets of E2F transcription factors, appeared to be the key determinants of Chemo responsiveness (Figure 2G). Collectively, our findings suggest that transcriptomic/proteomic signatures reflecting the biological consequences of high EGFR activation (i.e., EGFR dependency) have a predictive utility for determining EGFRi responsiveness that extends beyond mutation profiles alone.

### Prediction of CCR6 as a druggable target for EGFRi sensitivity

**Figure 3:**
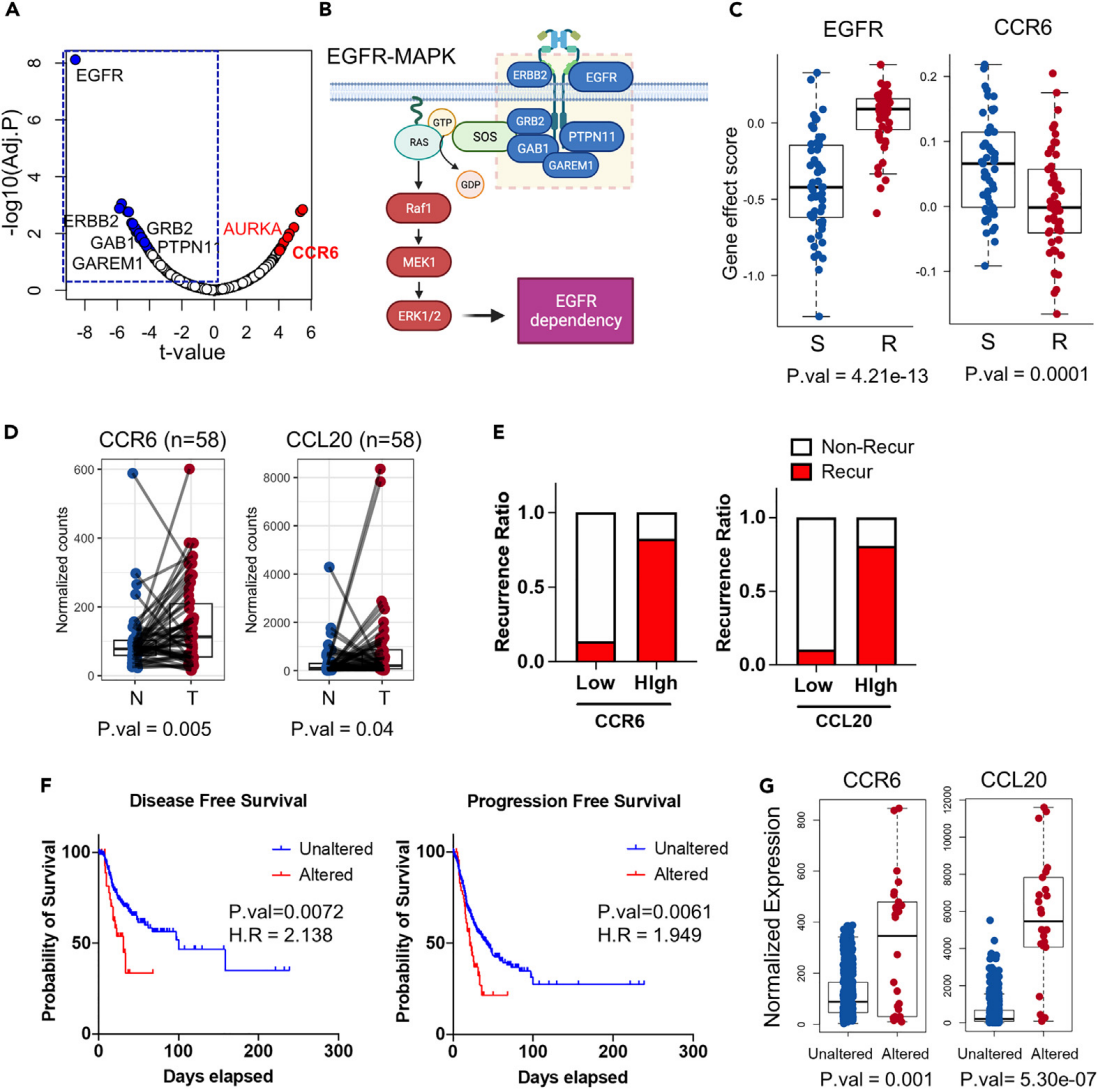
Derivation of CCR6 as a potential druggable target for EGFRi sensitivity (A) Volcano plot highlighting dependent genes in EGFRi-S (blue) or EGFRi-R (red) cell groups. Genes associated with the EGFR-MAPK axis are enclosed within a blue dotted box. (B) Schematic diagram illustrating the genes mediating and constituting the EGFR-MAPK signaling cascade. (C) Gene effect scores of EGFR (left) and CCR6 (right) in EGFRi-S and EGFRi-R cell groups. (D) Gene expression levels of CCR6 (left) and CCL20 (right) in matched tumor and normal pair samples from TCGA LUAD patient dataset. (E) Recurrence rates in patients categorized by CCR6 (left) and CCL20 (right) expression levels from the lung adenocarcinoma patients dataset.^41^ (F) Survival analysis showing disease-free and progression-free survival rates in patient groups with or without CCR6 alterations. (G) Gene expression level discrepancies for CCR6 (left) and CCL20 (right) in patient groups with or without CCR6 alterations.

The dependency on EGFR for cancer survival, which is a key determinant of EGFRi sensitivity, is also measurable using the Project Achilles dataset based on cell viability data following genetic perturbation.^39^ We focused on CCLs with sufficient drug response, genome, and transcriptome data (Figure S3A) and genes whose disruption results in a loss of viability in either the EGFRi-S or EGFRi-R cell groups (Figure S3B). The EGFRi-S cells exhibited a significant dependency on *EGFR, PTPN11, GRB2, GAB1*, *ERBB2,* and *GAREM1* (Figure 3A). Encouragingly, these genes encode key proteins that govern EGFR signaling and MAPK activation, leading to EGFR dependency (Figure 3B). Furthermore, EGFR was the most significant gene whose deficiency rendered EGFRi-S cells vulnerable (Figure 3C). The set of genes displaying high dependency in EGFRi-R cells (highlighted in red) have the potential to be chemically targeted to sensitize resistant cancer cells to EGFRi (Figure 3A). Of the top 20 genes in this set, we narrowed down our selection based on their druggability, i.e., their susceptibility to chemical perturbation (Figure S3C). In addition to Aurora kinase A (AURKA), a previously validated target for reversing EGFRi resistance,^40^ CCR6, a chemokine receptor, was also found to meet the criteria for consideration (Figure 3C).

CCR6 is a G protein-coupled receptor (GPCR) that mediates signals upon binding with chemokine C-C motif ligand 20 (CCL20), the sole ligand for CCR6 primarily found within immune cells.^42^ The hyper-activation of the CCL20–CCR6 axis leads to autoimmune disease, making CCR6 a promising therapeutic target to prevent this type of disease.^43^ Consequently, a diverse range of CCR6 antagonists are currently in development^44^^,^^45^ as anti-inflammatory drugs. Furthermore, emerging evidence has highlighted the involvement of the CCL20–CCR6 axis in cancer progression.^46^^,^^47^^,^^48^^,^^49^ Consistent with this, both CCR6 and CCL20 exhibited significant upregulation in tumor tissues compared to their matched normal counterparts in LUAD patients (Figure 3D). Elevated expression levels of CCR6 and CCL20 were associated with a higher recurrence ratio (Figure 3E). Moreover, survival analysis of LUAD patients revealed that individuals with sequence variation in CCR6 or CCL20 experienced shorter disease-free and progression-free intervals compared to those with wildtype CCR6 or CCL20 (Figure 3F). Notably, patient with alterations in CCR6 or CCL20 also exhibited higher expression levels of these genes compared to the unaltered group (Figure 3G). These findings collectively suggest that the upregulation of the CCR6–CCL20 axis may play a significant role in the progression of lung cancer, potentially leading to increased mortality rates.

### CCR6 inhibitor for sensitizing EGFR-resistant cancer cells to EGFRi

**Figure 4:**
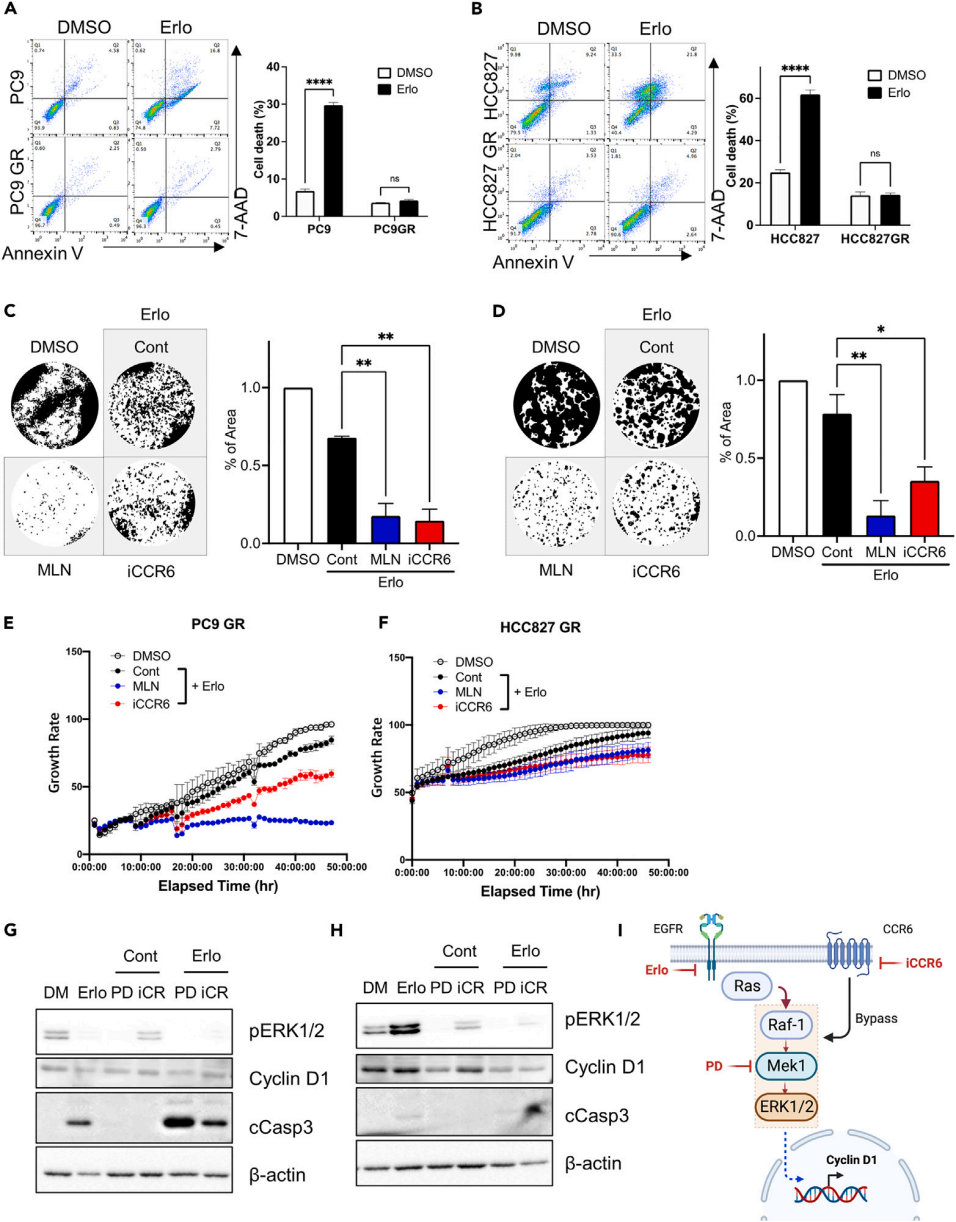
CCR6 inhibitor sensitizing lung cancer cells resistant to EGFRi (A and B) Flow cytometry for Annexin V and 7-AAD 48 h after treatment with 2 μM erlotinib (left) in (A) PC9, PC9 GR and (B) HCC827, HCC827 GR, with a graphical presentation of annexin V and 7-AAD positive cells (right). (C and D) Representative images of clonogenic assays after the indicated dose of the inhibitor with 2 μM erlotinib in (C) PC9 GR cells and (D) HCC827 GR cells. (E and F) Time-dependent proliferation of (E) PC9 GR and (F) HCC827 GR after inhibitor treatment with 2 μM erlotinib, 100 nM MLN8237, and 500 nM CCR6 inhibitor. (G and H) Immunoblotting analysis for cyclin D1, cleaved caspase3, and phospho-ERK [Thr202/Tyr204] on (G) PC9 and (H) PC9 GR after CCR6 inhibitor treatment with 2 μM erlotinib, 100 nM MLN8237, and 500 nM β-actin for equal protein loading. (I) Schematic diagram of the bypassing of EGFR signaling induced by the CCR6 axis. Data are presented as the mean ± standard deviation (SD) of independent experiments. Statistical significance was assessed using p value threshold of 0.05. Asterisks denote significance levels: ∗p < 0.05, ∗∗p < 0.01, ∗∗∗p < 0.001, ∗∗∗∗p < 0.0001; n.s. denotes non-significant.

To validate the effect of the chemical perturbation of CCR6 on EGFRi sensitivity, we employed PC9GR and HCC827GR, cell models previously established from PC9 and HCC827 cell lines, respectively, to exhibit acquired resistance to gefitinib.^50^ The EGFRi resistance of PC9GR and HCC827GR was confirmed by comparing the apoptotic population after erlotinib treatment to each parental cell line (Figures 4A and 4B). The chemical perturbation of the predicted protein targets was achieved via treatment with MLN8237 (MLN), a selective Aurora A inhibitor, or with a CCR6 antagonist (iCCR6).^51^ Clonogenic assays revealed that MLN and iCCR6 treatment clearly potentiated the anti-cancer effect of erlotinib in PC9GR (Figure 4C) and HCC827GR (Figure 4D). Live-cell images were used to monitor the time-dependent cell growth, showing that the anti-growth effect of erlotinib was significantly stronger with the inhibition of CCR6 (Figures 4E and 4F). The effect of erlotinib on the inhibition of EGFR-MAPK signaling was determined based on the level of ERK1/2 phosphorylation (pERK1/2). While erlotinib completely inhibited pERK1/2 in PC9 (Figure 4G), pERK1/2 levels were higher in PC9GR, indicating the presence of a bypass mechanism (Figure 4H). The high pERK1/2 levels following erlotinib treatment in PC9GR were markedly attenuated by CCR6 inhibition in a similar manner to MEK1 inhibition with PD-0325901 (PD), a potent MEK1 inhibitor (Figure 4H). The levels of cyclin D1 protein, a key regulator of cell cycle progression whose transcription is controlled by ERK1/2 activity,^52^ were suppressed by iCCR6 treatment with erlotinib (Figure 4H). Notably, the clinical approval of inhibitors targeting CDK4/6, whose activity is determined by cyclin D1 protein level, highlights the pronounced clinical significance of cyclin D1 in the context of cancer therapeutics, particularly for breast cancer.^53^ Unlike the strong apoptosis (determined by cleaved caspase 3 [cCasp3]) observed in PC9, no clear apoptosis was observed following CCR6 inhibition in PC9GR (Figures 4G and 4H). These data suggest that the substantial decrease in cell growth observed with the combined treatment of erlotinib and iCCR6 (Figures 4C and 4D) is primarily attributed to the suppression of cyclin D1 through the inhibition of ERK1/2 activity (Figure 4H). This outcome is likely due to the disruption of the CCR6-mediated bypass pathway to the MEK–ERK axis (Figure 4I), which causes cell-cycle arrest rather than apoptosis.

### Transcriptome profiles representing metabolic changes due to CCR6 inhibition

**Figure 5:**
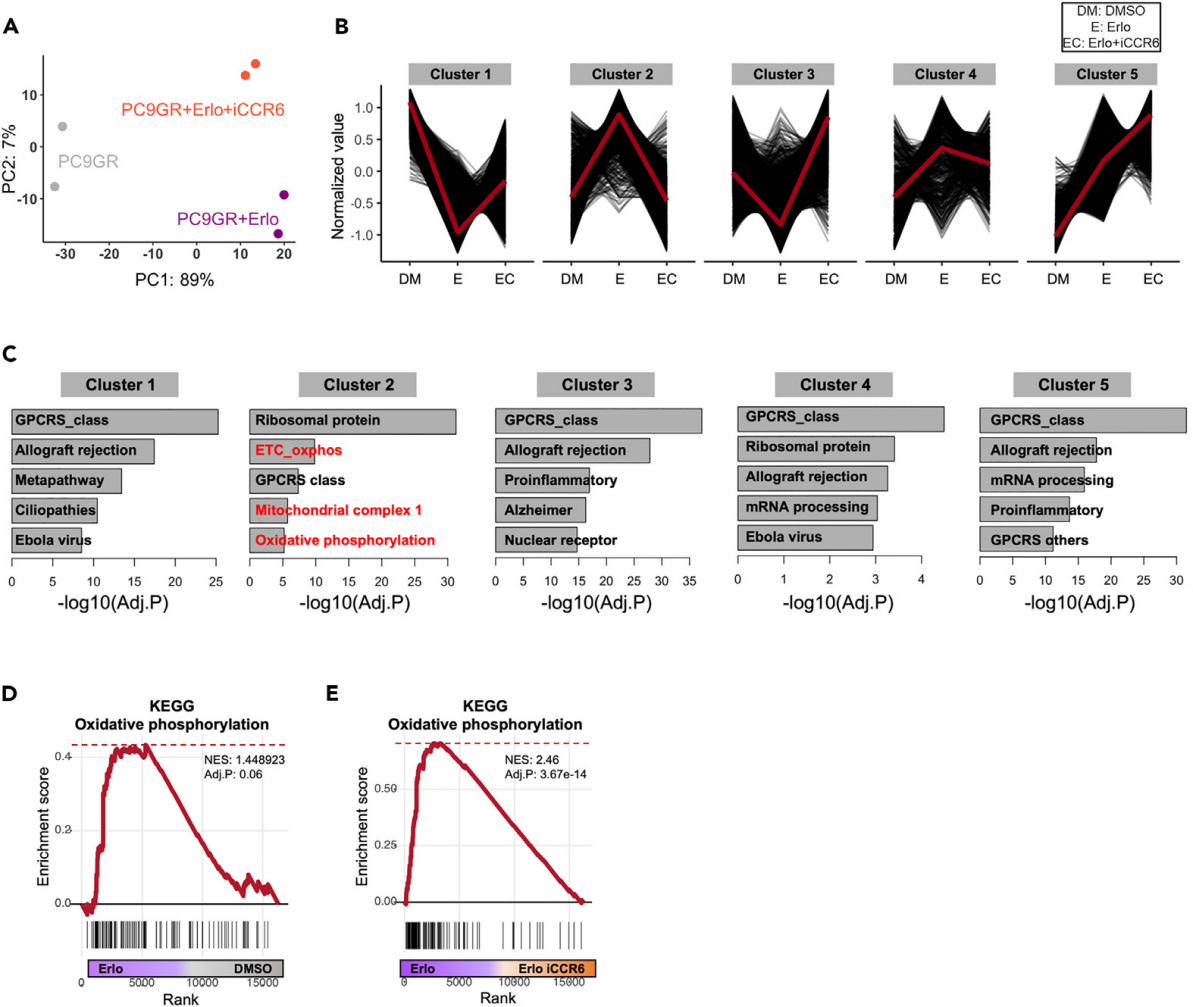
Transcriptome profiles capturing metabolic changes induced by CCR6 inhibition (A) Principal component analysis (PCA) of PC9 GR cells treated with DMSO (gray), erlotinib (purple), and erlotinib+iCCR6 (orange). (B) Hierarchical clustering results representing five gene clusters showing distinct transcriptomic changes following each treatment condition. 9C) The top five GO categories significantly enriched in each gene cluster. Gene sets related to oxidative phosphorylation are highlighted in red. (D and E) GSEA plots displaying the enrichment for a gene set “KEGG_oxidative_phosphorylation” in gene rank based on (D) differential expression between the erlotinib-treated group vs. DMSO-treated group and (E) differential expression between erlotinib-treated group vs. erlotinib/CCR6i-treated group.

To comprehensively assess the effects of CCR6 inhibition on PC9GR cells on a genome-scale, RNA sequencing was conducted on PC9GR cells involving three distinct perturbation conditions: vehicle only, iCCR6 alone, and a combination of iCCR6 and erlotinib. Principal component analysis of the resulting transcriptomic profiles revealed distinct patterns of gene expression within each perturbation group (Figure 5A). To investigate the perturbation-induced changes in the biological pathways, we employed hierarchical clustering to group genes based on their expression patterns across different perturbation conditions (Figure 5B). Subsequently, functional enrichment analysis was conducted on each gene cluster (Figure 5C; Table S3). Among the clusters, we focused on two specific clusters where the genes downregulated (cluster 1) or upregulated (cluster 2) following erlotinib treatment were reversed by co-treatment with iCCR6 (Figure 5B). Intriguingly, the cluster 2 genes were significantly associated with oxidative phosphorylation (OXPHOS)-related functions (Figure 5C). This observation aligns with the well-established characteristics of acquired therapy-resistant cancer cells, which often undergo a metabolic shift toward OXPHOS to maintain their growth.^54^^,^^55^ The PC9GR cells with strong EGFRi resistance (Figure 4A) exhibited the overall upregulation of OXPHOS-related genes following erlotinib treatment compared with the control (Figure 5D). Conversely, the significant repression of these genes was observed following co-treatment with iCCR6 (Figures 5E, S4A, and S4B). Taken together, these findings suggest that CCR6 inhibition has the potential to reverse acquired EGFRi resistance by modulating OXSPHOS.

### Attenuation of the metabolic shift via the inhibition of CCR6 and EGFR

**Figure 6:**
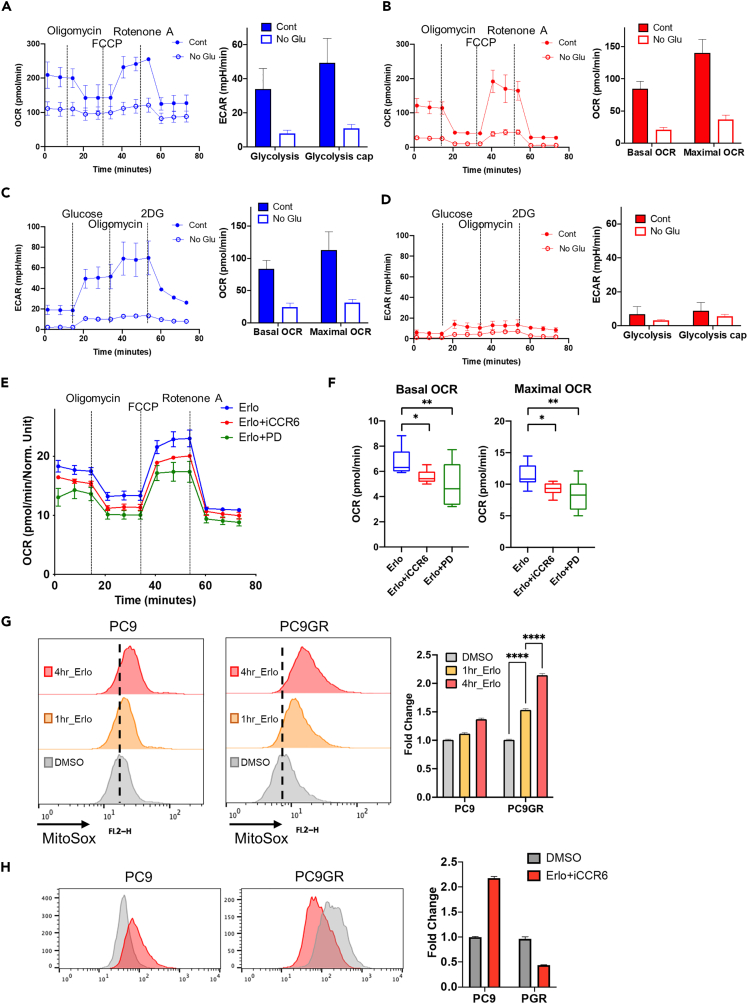
Metabolic shift by inhibiting both CCR6 and EGFR (A and B) Oxygen consumption rate (OCR) of (A) PC9 cells and (B) PC9 GR cells with and without glucose determined using the Seahorse XFe96 (OM: oligomycin; FCCP: trifluoromethoxy carbonylcyanide phenylhydrazone; R/A: Rotenone/Antimycin A) (left). Indicators of OXPHOS (basal respiration and maximal respiration) measured based on the assays from the left panels (right). (C and D) Extracellular acidification rate (ECAR) of (C) PC9 cells and (D) PC9 GR cells with and without glucose determined using the Seahorse XFe96 (Glc: glucose; Oligo: oligomycin; 2-DG: 2-deoxyglucose) (left). Indicators of glycolysis activity (glycolysis and glycolytic capacity) measured based on the assays from the left panels (right). (E) OCR of PC9 GR cells with an indicated dose of the inhibitor with 2 μM erlotinib determined using the Seahorse XFe96. (F) Indicators of OXPHOS (basal respiration and maximal respiration) measured based on the assays from (E). (G and H) Mitochondrial ROS production (G) after erlotinib treatment and (H) erlotinib/iCCR6 treatment in PC9 and PC9 GR cells determined using MitoSOX staining and following flow cytometry (left). The staining intensities were quantified by calculating fold change values (right). Data are presented as the mean ± standard deviation (SD) of independent expreiments. Statistical significance was assessed using p value threshold of 0.05. Asterisks denote significance levels: ∗p < 0.05, ∗∗p < 0.01, ∗∗∗p < 0.001, ∗∗∗∗p < 0.0001; n.s. denotes non-significant.

Because the transcriptome signature indicated a drastic metabolic shift following drug treatment of PC9GR cells, we measured the oxidative consumption rate (OCR) and extracellular acidification rate (ECAR), which correspond to the OXPHOS and the anaerobic glycolysis, respectively. When PC9 and PC9GR were subjected to glucose-free conditions, both PC9 (Figure 6A) and PC9GR (Figure 6B) experienced a significant reduction in the OCR. However, the ECAR, which was clearly suppressed by glucose-free supplementation in PC9 cells (Figure 6C) remained inactive in PC9GR, regardless of glucose level (Figure 6D). These results illustrated the high dependency of ATP production on OXPHOS in PC9GR cells. In support of the observed transcriptomic changes (Figure 5E), co-treatment using erlotinib with either iCCR6 or PD attenuated the OCR (Figure 6E), as well as basal and maximal OCR levels (Figure 6F). Of note, the elevated ATP production from OXPHOS is important for EGFRi resistance.^56^ Additionally, increased OXPHOS in the therapy-resistant cancer cells elevates ROS production,^55^ which has also been observed in EGFRi-R lung cancer cells.^57^ In order to readily quantify the mitochondrial activity after erlotinib treatment, mitochondrial ROS production was measured with MitoSOX as previously described.^55^ Consistently, the fold change in the MitoSOX-positive population was drastically increased in PC9GR cells compared with PC9 (Figure 6G), suggesting that higher mitochondrial activity (i.e., OXPHOS) following erlotinib treatment leads to EGFRi resistance. Interestingly, iCCR6 treatment was sufficient to abrogate the induction of mitochondrial activity after erlotinib treatment (Figure 6H). These results suggest that disrupting mitochondrial activity by inhibiting CCR6 has the potential to be a novel therapeutic approach to overcome EGFRi resistance.

## Discussion

EGFRi treatment is prescribed for cancer patients who harbor specific EGFR mutations that confer EGFR dependency, ensuring the efficacy of the treatment. To widen patient eligibility for EGFRi treatment, the relationship between EGFRi responsiveness and atypical mutations, i.e., those other than classical EGFR mutations, has been extensively characterized.^6^^,^^58^ Through integrated omics analysis of more than 1000 CCL datasets, transcriptomic and proteomic signatures (i.e., the estrogen response and P53 pathway) exhibited a highly significant positive correlation with EGFRi sensitivity, even in CCLs with wildtype EGFR. In a similar manner, the high dependency on gene products from the EGFR-MAPK pathway in EGFRi-S cells was revealed in the analysis of Achilles data. These results highlight that extensive CCL databases such as CCLE, CTRP, and Achilles are useful for identifying molecular signatures associated with cell susceptibility to specific drugs. Our further prediction suggested AURKA and CCR6 as putative druggable targets to perturb the growth and/or survival of EGFRi-R cells. This prediction was validated by biochemical experiments based on two isogenic lung cancer models with acquired EGFRi resistance. We found that active CCR6 signaling served as a key bypass signaling pathway for ERK1/2 during EGFRi treatment, thus simultaneous inhibition of EGFR and CCR6 uncoupled the signal toward ERK1/2 for cyclin D1 expression. The OXPHOS dependency for EGFRi resistance^56^ was also attenuated by the inhibition of CCR6.

Due to the limitation of CCLs to represent the complexity of real cancer, the biological characteristics of a few CCLs often fail to recapitulate cellular events and/or drug responses in actual cancers.^59^^,^^60^ This is why clinical datasets such as TCGA are widely used to support the clinical relevance of the findings from CCLs. Nevertheless, recent advances in computational methods leveraging large-scale omics data from CCLs have facilitated a deeper understanding of the diverse biological characteristics of cancers, as well as the prediction of drug responses.^22^^,^^61^^,^^62^ However, despite the numerous reports of *in silico* predictions that establish novel associations between molecular targets and drug responses, subsequent *in vitro* or *in vivo* biochemical analysis to validate these predictive outcomes has been lacking except for a few studies.^63^

In this context, our approach to identifying molecular signatures for EGFRi responsiveness to recapitulate EGFR dependency broadens the limited eligibility for EGFRi treatment, which has traditionally been based on oncogenic mutations. The estrogen response, an unexpected molecular signature for EGFRi responsiveness, corresponds to EGFR dependency considering the clear bidirectional signaling between ER and EGFR^35^ and the role of ER in EGFRi resistance.^64^^,^^65^ Thus, it is intriguing that a combined anti-cancer therapy of EGFRi with an ER antagonist has demonstrated promising clinical outcomes.^66^^,^^67^ The consequent analysis of Achilles data revealed CCR6 as a putative druggable target along with AURKA, the chemical inhibition of which would override EGFRi resistance.^40^ CCL20 is strongly promoted by EGFR signaling^49^ and contributes to MAPK activation in cancers.^49^^,^^68^ Thus, the CCR6 antagonist currently under clinical trial (phase I) developed for anti-inflammation activity (NCT04388878) could be a feasible option for combined treatment with EGFRi for EGFRi-resistant cancers.

### Conclusion

This study sought to broaden eligibility for EGFRi treatment by identifying both drug-response biomarkers linked to EGFRi sensitivity and therapeutic targets for EGFRi resistance. Transcriptomic and proteomic data indicated that the estrogen response signatures serve as promising predictors of EGFRi sensitivity, surpassing the conventional reliance on the EGFR mutational status as the sole response biomarker. Additionally, through the integration of genome-wide loss-of-function screening data with drug-response data, our research highlights CCR6 as a therapeutic target capable of reversing resistance to EGFRi through the modulation of glucose metabolism. Our data-driven approach presents a feasible and efficient approach for identifying biomarkers and therapeutic targets to combat resistance to EGFRi. This strategy is applicable not only to EGFRi but also to a range of other drugs listed in the DepMap database.

### Limitations of the study

This study leverages diverse cell lines to identify biomarkers for EGFRi response and resistance targets. However, its reliance on cell lines limits generalizability, as they may not fully recapitulate the complexity and heterogeneity of patient tumors. Further research using animal models or clinical trials is crucial to assess their potential for improving patient outcomes. Furthermore, although CCR6 inhibition demonstrated growth arrest in EGFRi-resistant cells, verification of cell death induction is essential to validate its therapeutic potential. Subsequent research should prioritize determining whether CCR6 inhibition induces cell death in EGFRi-resistant cells and identifying the underlying molecular mechanisms.

## STAR★Methods

### Key resources table

**Table:** 

REAGENT or RESOURCE	SOURCE	IDENTIFIER
**Antibodies**		

Rabbit polyclonal anti-p44/42 MAPK (Erk1/2) (Thr202/Tyr204)	Cell Signaling Technology	Cat#9101S (RRID: AB_331646)
Mouse monoclonal anti-CCNB1	Santa Cruz	SC-245 (RRID: AB_627338)
Mouse monoclonal anti-beta actin	Santa Cruz	SC-47778 (RRID: AB_626632)
Rabbit monoclonal anti- Cleaved Caspase-3	Cell Signaling Technology	Cat#9664S (RRID: AB_2070042)

**Chemicals, peptides, and recombinant proteins**		

CCR6 inhibitor	MedChem Express	Cat# HY-112701
PD0325901	Peprotech	Cat# 3911091

**Critical commercial assays**		

Seahorse XF Glycolytic Rate Assay Kit	Agilent	Cat# 103344–100
Seahorse XF Mitochondrial Respiration Assay Kit	Agilent	Cat# 103260–100
MitoSOX™ Mitochondrial Superoxide Indicators	Invitrogen	Cat# M36008

**Deposited data**		

Raw and analyzed RNA-seq data	This paper	GEO: GSE244730

**Experimental models: Cell lines**		

PC-9 cells	Laboratory stocks	RRID: CVCL_B260
HCC827 cells	Laboratory stocks	RRID: CVCL_2063

**Software and algorithms**		

ImageJ	National Institutes of Health	https://imagej.nih.gov/ij/
JuLI stage	NanoEntek	http://www.julistage.com
Seahorse XF Pro Analyzer	Agilent	https://www.agilent.com
R version 4.2.3	R Development Core Team	https://www.r-project.org/
FASTQC version 0.11.9	Babraham Bioinformatics Group	https://www.bioinformatics.babraham.ac.uk/projects/fastqc/
TrimGalore version 0.6.6	Babraham Bioinformatics Group	https://www.bioinformatics.babraham.ac.uk/projects/trim_galore/
STAR version 2.7.9a	Alexander Dobin et al.^69^	https://github.com/alexdobin/STAR
RSEM version 1.3.3	Bo Li et al.^70^	https://github.com/deweylab/RSEM
GraphPad Prism 9 version.4.2.3	GraphPad Software, Inc.	https://www.graphpad.com/features
FlowJo	BD Biosciences	https://www.bdbiosciences.com/en-us/products/software/flowjo-v10-software

**Other**		

DepMap portal	Open-source	https://depmap.org/portal/
GDSC	Open-source	https://www.cancerrxgene.org
TCGA	Open-source	https://gdc.cancer.gov/about-data/publications/pancanatlas
cBioPortal	Open-source	https://www.cbioportal.org
MsigDB	Open-source	https://www.gsea-msigdb.org/gsea/msigdb
DrugBank	Open-source	https://go.drugbank.com

### Resource availability

#### Lead contact

Further information and requests for resources and reagents should be directed to and will be fulfilled by the lead contact, Haeseung Lee (haeseung@pusan.ac.kr).

#### Materials availability

This study did not generate new unique reagents.

#### Data and code availability

• Datasets for human cancer cell lines are available on the DepMap portal (https://depmap.org/portal/, version 21Q3) and GDSC (https://www.cancerrxgene.org). RNA-seq data for human cancer patients are available on TCGA (https://gdc.cancer.gov/about-data/publications/pancanatlas) and clinical data for TCGA samples are available on cBioPortal (https://www.cbioportal.org). Gene sets from WikiPathway, hallmark, and gene ontology databases are available on MsigDB (https://www.gsea-msigdb.org/gsea/msigdb). Chemical structural information for EGFR inhibitors is available on DrugBank (https://go.drugbank.com). RNA-seq data of PC9GR following chemical perturbations were deposited at the Gene Expression Omnibus (https://www.ncbi.nlm.nih.gov/geo/) under accession number GSE244730.
• This paper does not report original code.
• Any additional information required to reanalyze the data reported in this paper is available from the lead contact upon request.

### Experimental model and study participant details

#### Cell lines

The lung cancer cell lines PC9 (RRID: CVCL_B260, gender: male) and HCC827 (RRID: CVCL_2063, gender: female), along with their respective gefitinib-derived acquired resistance models PC9GR and HCC827GR cells, were previously established.^50^ All cells were maintained in Roswell Park Memorial Institute (RPMI) 1640 Medium. Specifically, PC9 and HCC827 cells were cultured in RPMI supplemented with 10% (v/v) fetal bovine serum and gentamicin (50 μg/mL) at 37°C in a humidified atmosphere with 5% CO2. Similarly, PC9GR and HCC827GR cells were maintained under the same conditions, but with the addition of 1 μM gefitinib. Cells were grown to 80–90% confluency and passaged at the desired fraction every 2–4 days. All cell lines have been authenticated by routine mycoplasma contamination testing.

### Method details

#### Annexin-V & 7-AAD staining

To determine the population of dead cells, Annexin-V/7-AAD staining was carried out in accordance with the manufacturer’s instructions (559763, BD Pharmingen). Results were analyzed using a Becton-Dickinson FACS Calibur-1 with Cell-QUEST software.

#### Colony formation assay

Cells (20–100 cells/well) were seeded and cultured overnight. After 24 h of incubation, cells were pre-treated with 2 μM erlotinib, 500 nM iCCR6, and 100 nM MLN8237. Cells were cultured for 10–14 days and the cells were washed with phosphate-buffered saline (PBS), and continued incubation. Cells were fixed with 4% paraformaldehyde solution and stained with 0.1% crystal violet.

#### Immunoblotting

Cells were lysed with tissue lysis buffer supplemented with 0.2 mM sodium vanadate and a 1 mM protease inhibitor cocktail (Roche, Basel, Switzerland). Antibodies for cyclin B1 (#sc-245) and β-actin (#sc-47778) were purchased from Santa Cruz Biotechnology, while antibodies for pERK (#9101) and cleaved Caspse-3 (#9664S) were purchased from Cell Signaling Biotechnology.

#### Measurements of the extracellular acidification rate (ECAR) and oxygen consumption rate (OCR)

The ECAR and OCR were examined using a Seahorse XF Glycolytic Rate Assay Kit (103344–100, Seahorse Bioscience, North Billerica, MA, USA) and a Seahorse XF Mitochondrial Respiration Assay Kit (103260–100, Seahorse Bioscience) following the manufacturer’s protocols and analyzed with a Seahorse XFe 24 Extracellular Flux Analyzer (Seahorse Bioscience) as previously described.^71^ PC9 and PC9GR cells were pre-conditioned, either with or without glucose-containing media, for 4 h prior to assessing the ECAR and OCR. Erlotinib, iCCR6, and PD0325901 were exposed to glucose-enriched media for the same duration before evaluating the ECAR and OCR.

#### Detection of intracellular ROS

After 1 and 4 h of erlotinib and iCCR6 treatment, reactive oxygen species (ROS) levels were determined by incubating cells with 5 μM mitoSOX (Invitrogen, cat#. M36008) for 30 min at 37°C. The cells were washed twice in PBS and then trypsinized, after which the fluorescence was measured using fluorescence-activated cell sorting (FACS; excitation at 488 nm and emission at 580 nm for mitoSOX). Data were analyzed using Flowjo.

#### Cell proliferation

A JuLI stage (NanoEntek, Seoul, Korea) was used to measure the cell proliferation rate. After cell seeding, plates were loaded on the JuLI stage for 2 days. Time-dependent live images were obtained and then analyzed using JuLI-Stat software (NanoEntek, Seoul, Korea) to determine time-dependent growth potential according to the manufacturer’s protocol.

#### Collection of cancer cell line and primary tissue datasets

Human CCL datasets were acquired from the DepMap portal (https://depmap.org/portal/, version 21Q3), which includes omics (genome, transcriptome, proteome) and phenotypic (genome-wide loss-of-function screening and drug response) datasets for over 1000 CCLs derived from more than 30 cancer types (Figure 1). CCL drug response data for EGFRi treatments, including erlotinib, gefitinib, and lapatinib, were utilized from three independent databases, CTRP, PRISM, and GDSC. The area under the dose-response viability curve (AUC) was used as a measure of drug sensitivity, with a lower AUC indicating greater drug sensitivity. Information on gene mutations and their oncogenic effect annotated by OncoKB were obtained from cBioPortal (https://www.cbioportal.org). OncoKB is a precision oncology knowledge base that contains information about the effects and treatment implications of cancer variants.^32^ The mRNA and protein expression levels of a gene were reported using log2-transformed transcript per million (TPM) values with a pseudo-count of 1 and normalized protein quantitation levels,^72^ respectively.

RNA-seq data for patients with lung adenocarcinoma (LUAD) were obtained from the TCGA legacy gene expression dataset using the GDCquery function within the R package TCGAbiolinks. This dataset contains read counts and TPM values for a total of 19,919 protein-coding genes across 515 patient samples. Additional information regarding genetic alterations and clinical data for individual samples was sourced from cBioPortal (https://www.cbioportal.org/). To compare gene expression patterns between normal and tumor tissues in LUAD patients, we selected a cohort of 58 patients for whom RNA-seq data from matched normal tissue were available.

#### Differential expression analysis and functional enrichment

To analyze differential gene and protein expression between EGFRi-R and EGFRi-S groups, we employed Wald tests and moderated *t*-tests, which were implemented in the R package DESeq2 (v3.15) and limma (v3.54), respectively. Differentially expressed genes (DEGs) and proteins (DEPs) were selected based on a false discovery rate (FDR)-adjusted p-value less than 0.01 and an absolute fold-change greater than 2 as the cut-offs. To investigate signaling pathways associated with these DEGs and DEPs, a hypergeometric test was performed using MSigDB hallmark gene sets. The resulting FDR-adjusted p values were subsequently transformed into −log_10_(FDR) to score each pathway’s significance. To assign pathway activity scores for individual samples in CCLE, TCGA, and PDX models, the single-sample GSEA (ssGSEA), using a profile of TPM values or protein intensities for all genes as an input, was conducted through the R package gsva. Additionally, gene set enrichment analysis (GSEA) was conducted to yield pathways significantly overrepresented at the top or the bottom of ranked gene lists acquired from the differential gene expression analysis between various groups (e.g., EGFRi-R vs. EGFRi-S, erlotinib vs. DMSO, or erlotinib+CCR6 inhibitor vs. erlotinib). The GSEA was performed using the R package fgsea with the following parameters: minSize of 10, maxSize of 500, and nperm of 100,000. The pathway score was defined as the product of −log_10_(FDR) and the sign of the normalized enrichment score (NES) to directly reflect the statistical significance.

#### Evaluation of EGFR mutations and transcriptomic signatures in predicting EGFRi sensitivity

To assess the predictive performance of EGFR mutations and transcriptomic signatures for determining EGFRi sensitivity, we utilized CCLs datasets in two independent databases GDSC and PRISM. First, we assigned transcriptomic signature scores to each CCL based on the enrichment scores obtained through ssGSEA for the estrogen response (early and late), P53 pathway, and EMT. Next, as for the mutational status, we quantified scores based on the mutation class to which each specific mutation type belongs (Figure 1C). To make the mutational status, i.e., presence or absence of EGFR mutations, more quantifiable, we roughly converted it into an ordered value that aligned with the known oncogenic potential of each mutation type (Figure S2D). Finally, we categorized CCLs falling within the lowest deciles of the AUC as EGFRi-S cells and conducted a receiver operating characteristic (ROC) analysis. The area under the ROC curve (AUROC) served as a quantitative measure for the predictive accuracy of each prediction method, with a higher AUROC value indicating better predictive performance.

#### Gene dependency analysis with the Achilles dataset

To predict genes whose deficiency could potentially render EGFRi-resistant cells vulnerable, we investigated the genetic knock-out (KO) effects associated with drug responses across CCLs. Genetic KO effect scores were obtained from the ‘Achilles_gene_effect_CERES.csv’ file accessible through the DepMap portal, which contains the effect score for 18,119 genes across 902 CCLs. These scores, commonly known as dependency scores, reflect the importance of a gene for the survival of a specific cell. A more negative value suggests that a gene is essential for the survival of a particular cell, whereas a higher positive value indicates that the gene is less essential. Our analysis focused on 531 CCLs that contained drug response, genome, and transcriptome data. Within these CCLs, we selected EGFRi-sensitive (EGFRi-S) and EGFRi-resistant (EGFRi-R) subgroups based on their sensitivity to EGFRi using a 10% AUC threshold (Figure S3A). We then quantified the difference in the gene effect scores between EGFRi-R and EGFRi-S cells using Student’s *t*-tests for each gene. A low negative *t*-statistic indicates that a gene is essential for the survival of EGFRi-S cells, while a high positive *t*-statistic suggests that the gene is essential for the survival of EGFRi-R cells.

#### Assessment of clinical relevance of CCR6 in lung cancer patients

The clinical relevance of CCR6 in lung adenocarcinoma patients was assessed using two independent datasets, TCGA and a previously published study.^41^ Survival analysis was conducted on TCGA LUAD patients to determine differences in disease-free and recurrence-free survival times between individuals with or without genetic alterations in a gene of interest, specifically either CCR6 or CCL20 genes. These genetic alterations included mutations, amplifications, and deletions, with the necessary information retrieved from cBioPortal. To quantify the observed survival differences, we employed two statistical approaches, Cox proportional hazards regression analysis and the log rank test to produce the hazard ratio (HR) and p-value, respectively. All statistical computations were carried out using the R package survival (v3.4-0).

#### Generation and preprocessing of RNA sequencing data

Total RNA was isolated from PC9 and PC9GR cells in duplicate using Trizol according to the manufacturer’s instructions. For library construction, we used the TruSeq Stranded mRNA Library Prep Kit (Illumina, San Diego, CA). Briefly, the strand-specific protocol included the following steps: (1) strand cDNA synthesis, (2) strand synthesis using dUTPs instead of dTTPs, (3) end repair, A-tailing, and adaptor ligation, and (4) PCR amplification. Each library was then diluted to 8 p.m. for 76 cycles of paired-read sequencing (2 × 75 bp) on an Illumina NextSeq 500 following the manufacturer’s recommended protocol.

The quality of the resulting fastq-formatted read data was assessed using FastQC (v0.11.9). Adapter sequences and low-quality bases (Phred score <20) were eliminated using TrimGalore (v0.6.6). Trimmed reads were then aligned to the human reference genome (GRCh38) using the STAR aligner^69^ (v2.7.9a) with default parameters. Gene-level expression values, including TPM and read counts were quantified using RSEM^70^ (v1.3.3.) with human gene annotations (GRCh38.84). Of the 60,728 genes, 19,482 protein-coding genes were utilized for subsequent analysis. The fastq files and pre-processed data are available in the Gene Expression Omnibus (https://www.ncbi.nlm.nih.gov/geo/) under accession number GSE244730.

#### Gene clustering based on drug-induced transcriptional changes

To group genes based on their expression changes in response to chemical perturbations, including DMSO, erlotinib, or erlotinib combined with a CCR6 inhibitor, we employed hierarchical clustering with a predefined cluster number of K = 5. In this clustering process, we began by normalizing gene expression values across all samples using z-transformation. This step ensured that the gene expression values were on a comparable scale. Next, we quantified the dissimilarity between genes using the Euclidean distance metric. Finally, to form clusters, we applied the complete linkage method that considers the maximum distance between any two data points within each cluster. To further associate signaling pathways with genes in each cluster, a hypergeometric test was performed using Wikipathway gene sets.

### Quantification and statistical analysis

Quantitative data were expressed as the mean ± standard deviation (SD). Student’s unpaired two-tailed t-tests were employed to assess the statistical significance of the mean differences between the two groups unless otherwise specified. Paired t-tests were empolyed to assess the statistical significance of the mean difference betweeen paired samples of normal and tumor tissues. Differences in the distribution of AUC between two groups (e.g., EGFR mutation vs. EGFR wildtype) were assessed using Kolmogorov–Smirnov (K-S) tests. p-values less than 0.05 were considered statistically significant. Significance levels were stratified as p < 0.05 (∗), p < 0.01 (∗∗), p < 0.001 (∗∗∗), and p < 0.0001 (∗∗∗∗), with *n.s.* denoting non-significant. All statistical analyses and graphical visualizations were carried out using GraphPad Prism 9 (GraphPad Software, La Jolla, CA, USA) or R software (version 4.2.3).

## Supplemental information

Document S1. Figures S1–S4

Table S1. List of cancer cell lines harboring EGFR mutation included in CTRP, PRISM, and GDSC databases, related to Figure 1

Table S2. Results of functional enrichment analysis on differentially expressed genes (DEGs) or proteins (DEPs) between EGFRi-resistant (R) versus EGFRi-sensitive (S) cell groups, related to Figure 2

Table S3. Results of functional enrichment analysis on five gene clusters showing distinct expression patterns following chemical perturbations in PC9GR cells, related to Figure 5
