# Supplementary material for: Systematic omics analysis identifies CCR6 as a therapeutic target to overcome cancer resistance to EGFR inhibitors
Source: iScience. 2024 Mar 7;27(4):109448. doi: 10.1016/j.isci.2024.109448 (PMC10972824; doi:10.1016/j.isci.2024.109448)
Supplement: Document S1. Figures S1–S4 [file mmc1.pdf]

iScience, Volume *27*

Supplemental information

Systematic omics analysis identifies CCR6 as a therapeutic target to overcome cancer resistance to EGFR inhibitors

Eun-Ji Kwon, Hyuk-Jin Cha, and Haeseung Lee

Figures S1 – S4

Figure S1 | EGFR inhibitor responsiveness across various CCL and their EGFR mutation status, related to Figure 1

Figure S2 | Omics features associated with EGFRi responsiveness, related to Figure 2

Figure S3 | Prediction of essential genes in EGFRi-S and EGFRi-R cell groups, related to Figure 3

Figure S4 | Enrichment of gene sets in the erlotinib-treated group compared to the DMSO-treated group, related to Figures 5

Figure S1

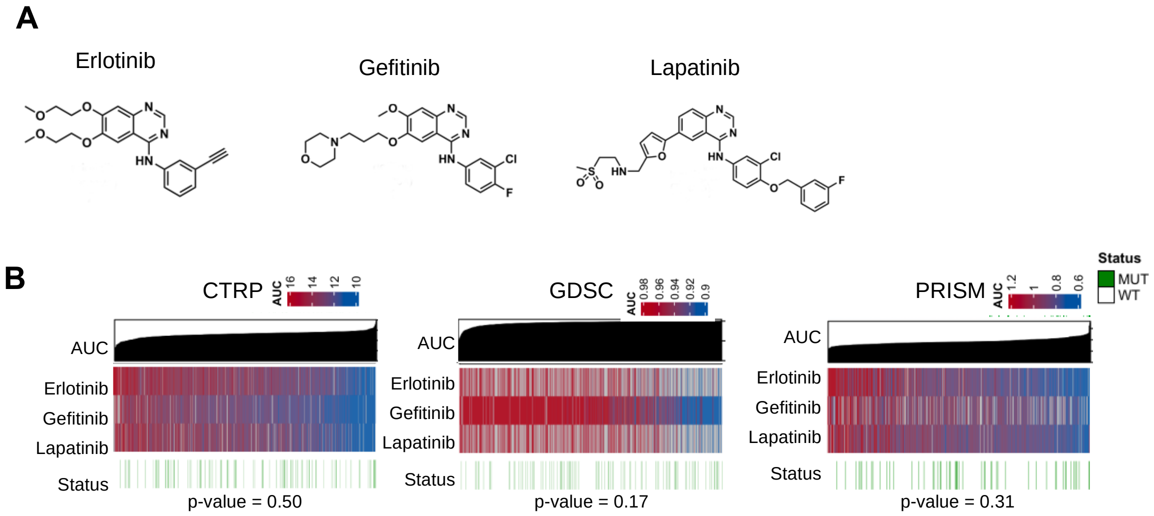

Figure S1. EGFR inhibitor responsiveness across various CCL and their EGFR mutation status. A) Chemical structures of three EGFRi, erlotinib, gefitinib, and lapatinib. **B)** Heatmaps showing EGFRi responsiveness AUC values across CCL and their EGFR mutation status, as observed in the CTRP, PRISM, and GDSC databases. CCLs are sorted in descending order based on the mean AUC values of three EGFRi. The statistical significance of the differences in AUC values between the groups of CCLs with and without EGFR mutations was assessed using K-S tests.

Figure S2

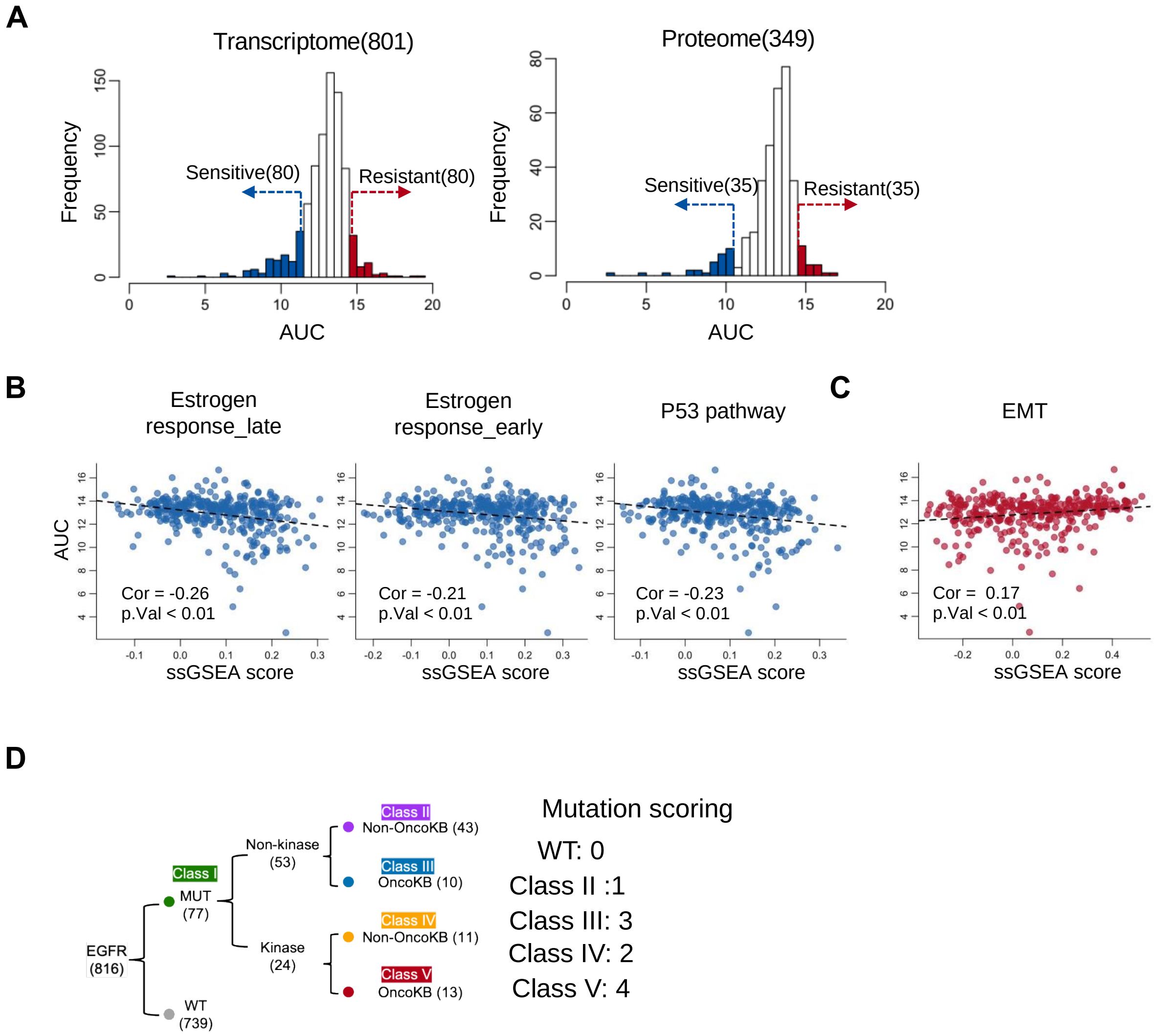

Figure S2. Omics features associated with EGFRi responsiveness. A) Distribution of EGFRi responsiveness AUC values of CCLs with substantial transcriptome (left) and proteome (right) datasets in CTRP. The lowest 10% and highest 10% of AUC values are highlighted in blue and red, respectively. **B,C)** Association between drug response AUC values and proteomic signatures for (B) the estrogen response (early and late) P53 pathway, and (C) EMT signature scores across CCLs. **D)** Categorization of EGFR mutation classes based on their location and oncogenic effect (left). A mutation score was assigned to each CCL, considering the known oncogenic potential of each mutation type it harbored (right).

Figure S3

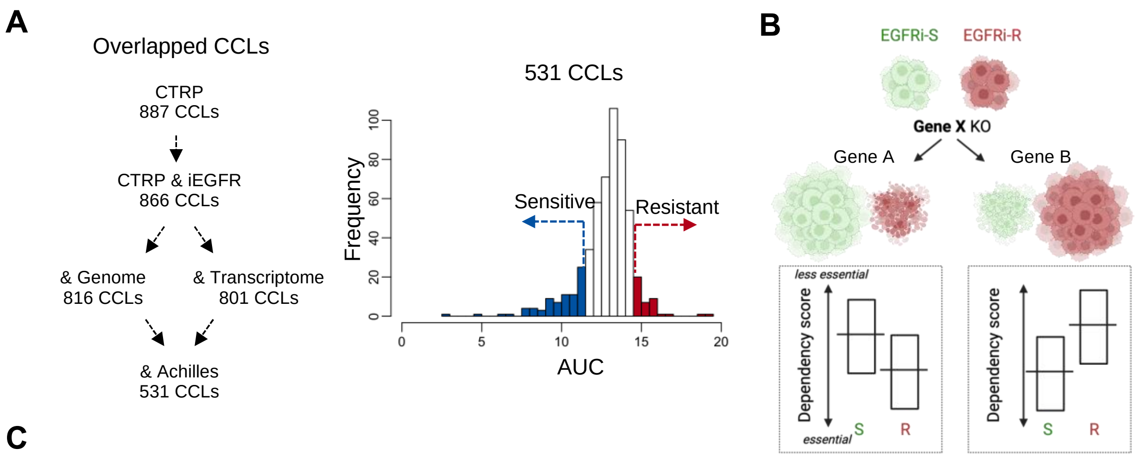

C

Gene	Description	Druggability
ORC6	Origin Recognition Complex Subunit 6	
CHMP4B	Charged multivesicular body protein 4B	
CDS2	CDP-Diacylglycerol Synthase 2	
FERMT2	FERM Domain Containing Kindlin 2	
PRKAR1A	Protein kinase cAMP-dependent type I regulatory subunit alpha	
CCT6A	Chaperonin Containing TCP1 Subunit 6A	
OR8B12	Olfactory receptor family 8 subfamily B member 12	
ERRFI1	ERBB receptor feedback inhibitor 1	
DOCK7	Dedicator of cytokinesis 7	
RFC3	Replication factor C subunit 3	
UBALD1	UBA like domain containing 1	
TMBIM1	Transmembrane BAX inhibitor motif containing 1	
POLE2	DNA polymerase epsilon 2, accessory subunit	
SNX25	Sorting nexin 25	
AURKA	Aurora kinase A	O
LDB1	LIM domain binding 1	
NAA50	N-alpha-acetyltransferase 50	
AUTS2	Activator of transcription and developmental regulator	
CCR6	C-C motif chemokine receptor 6	O
EIF3D	Eukaryotic translation initiation factor 3 subunit D	

Figure S3. Prediction of essential genes in EGFRi-S and EGFRi-R cell groups. A) Schematic diagram depicting the number of CCL used for the prediction of essential genes in EGFRi-S and EGFRi-R cell groups (left). Distribution of EGFRi responsiveness AUC values of 531 CCLs (right). The lowest 10% and highest 10% of AUC values are highlighted in blue and red, respectively. **B)** Schematic illustration showing the method for identifying genes exhibiting significant dependency differences between the EGFRi-S and EGFRi-R groups.

Gene A displayed high dependency in EGFRi-R cells, while Gene B exhibited high dependency in EGFRi-S cells. **C)** List of genes showing significant dependency in the EGFRi-R cell group in Fig. 3B.

Figure S4

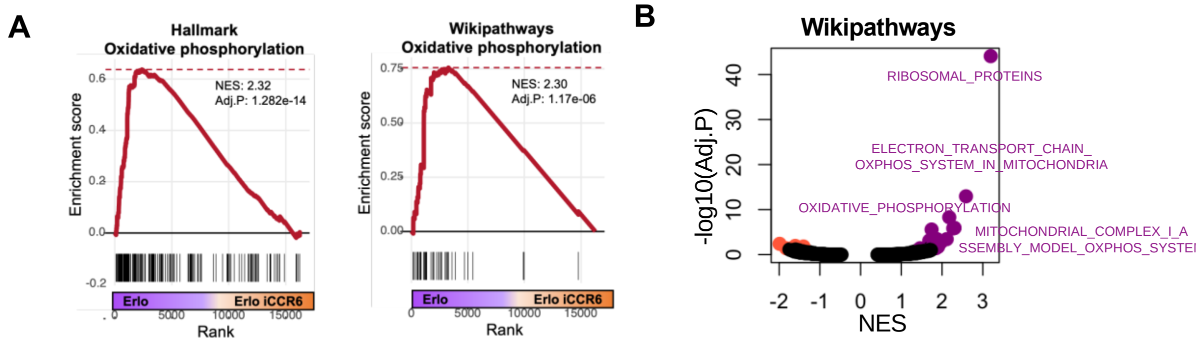

Figure S4. Enrichment of gene sets in the erlotinib-treated group compared to the DMSO-treated group. A) GSEA plots showing the enrichment of gene sets “Hallmark_oxidative_phosphorylation” (left) and “Wikipathways_oxidative_phosphorylation” (right) in gene rank based on differential expression between the erlotinib-treated group in comparison with the DMSO-treated group. **B)** A volcano plot depicting the result of GSEA using WikiPathways signatures when comparing the erlotinib-treated group with the erlotinib/CCR6i-treated group.
